# Cross-reactivity profiles of hybrid capture II, cobas, and APTIMA human papillomavirus assays: split-sample study

**DOI:** 10.1186/s12885-016-2518-4

**Published:** 2016-07-20

**Authors:** Sarah Preisler, Matejka Rebolj, Ditte Møller Ejegod, Elsebeth Lynge, Carsten Rygaard, Jesper Bonde

**Affiliations:** Clinical Research Centre and Department of Pathology, Copenhagen University Hospital Hvidovre, Kettegård Allé 30, 2650 Hvidovre, Denmark; Department of Pathology, Copenhagen University Hospital Hvidovre, Kettegård Allé 30, 2650 Hvidovre, Denmark; Department of Public Health, University of Copenhagen, Øster Farimagsgade 5, 1014 Copenhagen K, Denmark

**Keywords:** Human papillomavirus, Cervical cancer, HPV assays, Cross-reactivity, Clinical performance, Mass screening

## Abstract

**Background:**

High-risk Human Papillomavirus (HPV) testing is replacing cytology in cervical cancer screening as it is more sensitive for preinvasive cervical lesions. However, the bottleneck of HPV testing is the many false positive test results (positive tests without cervical lesions). Here, we evaluated to what extent these can be explained by cross-reactivity, i.e. positive test results without evidence of high-risk HPV genotypes. The patterns of cross-reactivity have been thoroughly studied for hybrid capture II (HC2) but not yet for newer HPV assays although the manufacturers claimed no or limited frequency of cross-reactivity. In this independent study we evaluated the frequency of cross-reactivity for HC2, cobas, and APTIMA assays.

**Methods:**

Consecutive routine cervical screening samples from 5022 Danish women, including 2859 from women attending primary screening, were tested with the three evaluated DNA and mRNA HPV assays. Genotyping was undertaken using CLART HPV2 assay, individually detecting 35 genotypes. The presence or absence of cervical lesions was determined with histological examinations; women with abnormal cytology were managed as per routine recommendations; those with normal cytology and positive high-risk HPV test results were invited for repeated testing in 18 months.

**Results:**

Cross-reactivity to low-risk genotypes was detected in 109 (2.2 %) out of 5022 samples on HC2, 62 (1.2 %) on cobas, and 35 (0.7 %) on APTIMA with only 10 of the samples cross-reacting on all 3 assays. None of the 35 genotypes was detected in 49 (1.0 %), 162 (3.2 %), and 56 (1.1 %) samples, respectively. In primary screening at age 30 to 65 years (*n* = 2859), samples of 72 (25 %) out of 289 with high-risk infections on HC2 and < CIN2 histology were due to cross-reactivity. On cobas, this was 106 (26 %) out of 415, and on APTIMA 48 (21 %) out of 224.

**Conclusions:**

Despite manufacturer claims, all three assays showed cross-reactivity. In primary cervical screening at age ≥30 years, cross-reactivity accounted for about one quarter of false positive test results regardless of the assay. Cross-reactivity should be addressed in EU tenders, as this primarily technical shortcoming imposes additional costs on the screening programmes.

## Background

High-risk human papillomavirus (HPV) is a necessary cause of cervical cancer. HPV testing is currently widely used for triage of women with cytological abnormalities i.e. atypical squamous cells of undetermined significance (ASCUS) and as a test of cure [1, 2]. In European countries including Norway, the Netherlands, Italy, Spain, Denmark, and Sweden primary HPV-based cervical screening is being piloted or a full-scale roll out is planned. In the USA, primary screening is at present undertaken as co-testing using cytology and HPV testing, but new recommendations advocate stand-alone HPV testing [3]. The role of HPV testing in screening is supported by the objectivity of test result read-outs and an improved protection of women from developing cervical cancer compared to cytology [4]. However, it is less specific for disease because most HPV infections clear spontaneously without leading to abnormalities. This means that false-positive test results, and the associated unnecessary diagnostic procedures, are common.

More than 100 HPV genotypes have been identified, of which 13 are high-risk (16, 18, 31, 33, 35, 39, 45, 51, 52, 56, 58, 59, and 68) [5]. Cross-reactivity of HPV assays to untargeted, low-risk (non-oncogenic), genotypes has been considered as a possible cause of false-positive HPV test results.

Cross-reactivity has only been systematically and independently evaluated for the most widely used assay, hybrid capture II (HC2), where it was most frequently due to low-risk genotypes 53, 66, and 70 [6–11]. The intensity of the positive signal in cross-reacting samples tended to be relatively weak [7, 8, 10], and the likelihood of cross-reactivity increased in multiple low-risk infections [6]. Most importantly, cross-reacting samples were rarely associated with high-grade cervical intraepithelial neoplasia (CIN) [6–8, 10]—clearly showing that cross-reactivity contributes to false-positive test results.

For more recently introduced commercially available assays, cross-reactivity profiles have not been independently established. Based on the data from the Danish Horizon study, we evaluated the frequency of cross-reactivity for HC2, cobas, and APTIMA in a large split-sample study.

## Methods

### Setting

In Denmark, women aged 23–65 years are invited for cytology-based cervical screening every three (age <50 years) or every 5 years (≥50 years). The design of the Horizon study was described in detail previously [12–17]. In short, consecutive SurePath samples from 5034 women evaluated at the Department of Pathology, Copenhagen University Hospital, Hvidovre, were tested with HC2, cobas, and APTIMA, and genotyped by CLART HPV2 Assay (Genomica, Madrid, Spain). All SurePath cytology was read under routine conditions following the Bethesda 2011 system using FocalPoint Slide Profiler and Imaging systems. Women with abnormal cytology (≥ASCUS) were managed according to routine screening recommendations. Women with normal cytology and a positive test result on at least one of the four HPV assays were additionally invited in 1.5 year for repeated cytology and HPV testing. For each woman, the worst histological diagnosis until December 2013, i.e. in approximately 2.5 years after the baseline testing, was retrieved from the nationwide Danish Pathology Data Bank (Patobank) [18].

### HPV testing

Cytology post-quot material was used for HC2 testing. The remaining HPV testing was undertaken on the original residual material diluted with SurePath (approximately 1:1). All testing was undertaken in strict concordance with the protocols issued and agreed upon with the manufacturers. The instrumentation was supplied and maintained by the manufacturers. Cut-offs for positive test results were set by the manufacturers: RLU/CO ≥1.0 for HC2; CT ≤40.5, ≤40.0, and ≤40.0 for cobas’s channels 16, 18, and other high-risk, respectively; and S/CO ≥0.5 for APTIMA.

CLART was used as the full genotyping reference assay. This *L1* DNA PCR assay reports 35 genotypes individually, including the 13 high-risk and 22 low-risk (6, 11, 26, 40, 42, 43, 44, 53, 54, 61, 62, 66, 70, 71, 72, 73, 81, 82, 83, 84, 85, and 89). It uses modified PGMY09/11 primers with amplification products hybridized onto a low density microarray. The amplified viral sequences are approximately 465 base pairs (bp) long, dependent on the genotype. Visualization was performed and thereafter automatically read on the CLART array reader (Genomica). A spiked rhCFTR plasmid is used as process control, while a DNA control of the human CFTR gene validates material sufficiency.

HC2 detects, collectively, the 13 high-risk HPV genotypes. The assay is based on hybridisation of viral DNA to a high-risk RNA probe cocktail. No retest range was used. Cobas is a real-time PCR analysis detecting the 13 high-risk genotypes plus genotype 66. The assay separately identifies genotypes 16 and 18, while the remaining 12 are detected collectively (“other high-risk”). The amplicons are approximately 165 bp long. APTIMA detects *E6/E7* mRNA expression of the 13 high-risk genotypes plus genotype 66 collectively using transcription-mediated amplification (TMA).

### Statistical analysis

Cross-reacting samples were defined as those with positive test results without evidence of high-risk HPV genotypes by CLART. A sample cross-reacting to low-risk genotypes was defined as one with a positive test result in which CLART detected only genotypes not targeted by the evaluated assay. This means that for HC2, cross-reactivity to low-risk genotypes was measured for 22 genotypes including genotype 66. For cobas and APTIMA assays, evaluation was undertaken for 21 genotypes, as they are both designed to detect genotype 66. Samples with a positive test result cross-reacting to unconfirmed genotypes, defined as non-CLART genotypes, were included in the analysis but evaluated separately [7, 8, 11]. From 5034 samples, 12 were invalid on CLART, reducing the number of eligible samples to 5022.

Assay-specific absolute cross-reactivity was defined as the proportion of cross-reacting samples among all studied samples, and assay-specific relative cross-reactivity as the proportion of cross-reacting samples among all samples with a positive test result. Genotypes most frequently involved in cross-reactivity were determined based on the distributions in single infections.

We used signal strength as a relative indicator of the amount of the viral target input material, and described its distribution with the median and interquartile range. If cobas returned a positive test result on more than one channel, the channel with the strongest signal was included in the analysis.

False-positive samples were defined as samples with a positive test result that were not followed by a diagnosis of ≥ CIN2. The origin of the samples was defined as primary screening or referral population using information on the women’s testing histories registered in the Patobank since January 2000. Referral population samples (*n* = 887) were defined as either primary screening samples showing abnormal cytology at any age, or as samples with a recent abnormality, regardless of age and cytology. A recent abnormality was defined as a preceding cervical cancer diagnosis, a histological CIN diagnosis in ≤3 years, cytological low-grade squamous intraepithelial lesions (LSIL) or worse, inadequate cytology, or a positive HPV test result in ≤12 months, and less abnormal cervical cytological or histological diagnoses in ≤15 months. Samples without a recent abnormality were predominantly screening samples. Since HPV screening has been discouraged for younger women [19], the primary screening population was restricted to 30–65 years (*n* = 2859). Cross-reactivity was compared between different groups by calculating relative proportions and their 95 % confidence intervals by assuming lognormal distribution.

## Results

### Cross-reactivity by assay

Among 5022 unselected samples (range: 16–89 years, mean = 37.3, SD = 12.3, 4748 (95 %) 23–65 years), 1262 (25 %) had at least one of the 13 high-risk genotypes detected by CLART, and 1333 (27 %) when genotype 66 was included. Furthermore, 1024 (20 %) were positive on HC2, 1345 (27 %) on cobas, and 838 (17 %) on APTIMA (Table 1). Of these, CLART detected only low-risk genotypes in 109 samples for HC2, 62 for cobas, and 35 for APTIMA. Acknowledging that cross-reactivity was assessed based on one more genotype for HC2 than for cobas and APTIMA (genotype 66), absolute cross-reactivity to low-risk genotypes was 2.2, 1.2, and 0.7 %, respectively, and relative cross-reactivity was 10.6, 4.6, and 4.2 %, respectively. Absolute cross-reactivity to low-risk genotypes was significantly higher for HC2 than for the other assays, and that of cobas was significantly higher compared to APTIMA. Relative cross-reactivity to low-risk genotypes was significantly higher for HC2, and statistically similar between cobas and APTIMA.

**Table 1:** Samples cross-reacting to low-risk and unconfirmed genotypes

	Total (any of the three assays)	HC2	cobas	APTIMA
Overall				
Positive test results, Total population (*N* = 5022)	1505	1024 (20.4 %)	1345 (26.8 %)^a^	838 (16.7 %)^a^
Positive test results, Primary screening population age 30–65 years (*N* = 2859)	553	335 (11.7 %)	464 (16.2 %)	270 (9.4 %)
Positive test results, Referral population (*N* = 887)	499	401 (45.2 %)	453 (51.1 %)	332 (37.4 %)
Cross-reactivity to low-risk genotypes				
Total population (*N* = 5022)				
Cross-reacting samples	157	109	62	35
Absolute cross-reactivity	–	109/5022 (2.2 %)	62/5022 (1.2 %)	35/5022 (0.7 %)
Absolute cross-reactivity (vs. HC2)	–	1 (reference)	0.6 (0.4 to 0.8)	0.3 (0.2 to 0.5)
Relative cross-reactivity	–	109/1024 (10.6 %)	62/1345 (4.6 %)	35/838 (4.2 %)
Relative cross-reactivity (vs. HC2)	–	1 (reference)	0.4 (0.3 to 0.6)	0.4 (0.3 to 0.6)
Primary screening population, age 30–65 years (*N* = 2859)				
Cross-reacting samples	61	43	20	13
Absolute cross-reactivity	–	43/2859 (1.5 %)	20/2859 (0.7 %)	13/2859 (0.5 %)
Absolute cross-reactivity (vs. HC2)	–	1 (reference)	0.5 (0.3 to 0.8)	0.3 (0.2 to 0.6)
Relative cross-reactivity	–	43/335 (12.8 %)	20/464 (4.3 %)	13/270 (4.8 %)
Relative cross-reactivity (vs. HC2)	–	1 (reference)	0.3 (0.2 to 0.6)	0.4 (0.2 to 0.7)
Referral population (*N* = 887)				
Cross-reacting samples	58	47	16	8
Absolute cross-reactivity	–	47/887 (5.3 %)	16/887 (1.8 %)	8/887 (0.9 %)
Absolute cross-reactivity (vs. HC2)	–	1 (reference)	0.3 (0.2 to 0.6)	0.2 (0.1 to 0.4)
Relative cross-reactivity	–	47/401 (11.7 %)	16/453 (3.5 %)	8/332 (2.4 %)
Relative cross-reactivity (vs. HC2)	–	1 (reference)	0.3 (0.2 to 0.5)	0.21 (0.1 to 0.4)
Primary screening vs. referral population				
Absolute cross-reactivity (95 % confidence interval)	–	0.3 (0.2 to 0.4)	0.4 (0.2 to 0.7)	0.5 (0.2 to 1.2)
Relative cross-reactivity (95 % confidence interval)	–	1.1 (0.7 to 1.6)	1.2 (0.6 to 2.3)	2.0 (0.8 to 4.8)
Cross-reactivity to unconfirmed genotypes				
Total population (*N* = 5022)				
Cross-reacting samples	223	49	162	56
Absolute cross-reactivity	–	49/5022 (1.0 %)	162/5022 (3.2 %)	56/5022 (1.1 %)
Absolute cross-reactivity (vs. HC2)	–	1 (reference)	3.3 (2.4 to 4.5)	1.1 (0.8 to 1.7)
Relative cross-reactivity	–	49/1024 (4.8 %)	162/1345 (12.0 %)	56/838 (6.7 %)
Relative cross-reactivity (vs. HC2)	–	1 (reference)	2.5 (1.8 to 3.4)	1.4 (1.0 to 2.0)
Primary screening population, age 30–65 years (*N* = 2859)				
Total positive test results	553	335 (11.7 %)	464 (16.2 %)	270 (9.4 %)
Cross-reacting samples	126	30	87	36
Absolute cross-reactivity	–	30/2859 (1.0 %)	87/2859 (3.0 %)	36/2859 (1.3 %)
Absolute cross-reactivity (vs. HC2)	–	1 (reference)	2.9 (1.9 to 4.4)	1.2 (0.7 to 1.9)
Relative cross-reactivity	–	30/335 (9.0 %)	87/464 (18.8 %)	36/270 (13.3 %)
Relative cross-reactivity (vs. HC2)	–	1 (reference)	2.1 (1.4 to 3.1)	1.5 (0.9 to 2.4)
Referral population (*N* = 887)				
Total positive test results	499	401 (45.2 %)	453 (51.1 %)	332 (37.4 %)
Cross-reacting samples	37	9	27	8
Absolute cross-reactivity	–	9/887 (1.0 %)	27/887 (3.0 %)	8/887 (0.9 %)
Absolute cross-reactivity (vs. HC2)	–	1 (reference)	3.0 (1.4 to 6.3)	0.9 (0.3 to 2.3)
Relative cross-reactivity	–	9/401 (2.2 %)	27/453 (6.0 %)	8/332 (2.4 %)
Relative cross–reactivity (vs. HC2)	–	1 (reference)	2.3 (1.3 to 5.6)	1.1 (0.4 to 2.8)
Primary screening vs. referral population				
Absolute cross-reactivity (95 % confidence interval)	–	1.0 (0.5 to 2.2)	1.0 (0.7 to 1.5)	1.4 (0.7 to 3.0)
Relative cross-reactivity (95 % confidence interval)	–	4.0 (1.9 to 8.3)	3.1 (2.1 to 4.7)	5.5 (2.6 to 11.7)

^a^Genotype 66 was the only detected genotype among those that are targeted by cobas or APTIMA in 31 (2.3 %) and 11 (1.3 %), respectively, of the samples with positive test results

Absolute cross-reactivity to unconfirmed genotypes was 1.0 % on HC2, 3.2 % on cobas, and 1.1 % on APTIMA, and relative cross-reactivity was 4.8, 12.0, and 6.7 %, respectively. Absolute cross-reactivity to unconfirmed genotypes was significantly higher for cobas than for the other two assays.

Cross-reactivity to low-risk genotypes was more frequent in younger women and in abnormal cytology (Table 2). Cross-reactivity to unconfirmed genotypes did not show a trend by age, but was, for cobas and APTIMA, somewhat more frequent in normal cytology.

**Table 2:** Characteristics of cross-reacting samples in 5022 women

	Total (any of the three assays)	HC2	cobas	APTIMA
Cross-reactivity to low-risk genotypes				
Age				
<30 years (*n* = 1683)	75	51 (3.0 %)	34 (2.0 %)	15 (0.9 %)
≥30 years (*n* = 3339)	82	58 (1.7 %)	28 (0.8 %)	20 (0.6 %)
Concurrent cytology				
Normal (*n* = 4630)	116	71 (1.5 %)	56 (1.2 %)	29 (0.6 %)
Abnormal (*n* = 367)	40	37 (10.1 %)	6 (1.6 %)	6 (1.6 %)
Inadequate (*n* = 25)	1	1 (4.0 %)	0 (0.0 %)	0 (0.0 %)
Histology outcome				
CIN2 (*n* = 60)	2	2 (3.3 %)	0 (0.0 %)	1 (1.7 %)
CIN3 or worse (*n* = 118)^a^	4	4 (3.4 %)	2 (1.7 %)	1 (0.8 %)
Cross-reactivity to unconfirmed genotypes				
Age				
<30 years (*n* = 1683)	73	15 (0.9 %)	56 (3.3 %)	15 (0.9 %)
≥30 years (*n* = 3339)	150	34 (1.0 %)	106 (3.2 %)	41 (1.2 %)
Concurrent cytology				
Normal (*n* = 4630)	214	44 (1.0 %)	157 (3.4 %)	54 (1.2 %)
Abnormal (*n* = 367)	7	4 (1.1 %)	3 (0.8 %)	2 (0.5 %)
Inadequate (*n* = 25)	2	1 (4.0 %)	2 (8.0 %)	0 (0.0 %)
Histology outcome				
CIN2 (*n* = 60)	0	0 (0.0 %)	0 (0.0 %)	0 (0.0 %)
CIN3 or worse (*n* = 118)^a^	1	1 (0.8 %)	1 (0.8 %)	1 (0.8 %)

^a^Of which three cases of cervical cancer

Relative cross-reactivity to low-risk genotypes on all three assays was not significantly different in the referral compared to the primary screening population. Absolute cross-reactivity to low-risk genotypes, however, was significantly lower on HC2 and cobas in the screening than in the referral population. This is probably a reflection of a lower HPV prevalence in primary screening. The patterns were different for cross-reactivity to unconfirmed genotypes, with relative cross-reactivity being more frequent in the screening than in the referral population (Table 1).

### Cross-reactivity concordance?

**Fig. 1:**
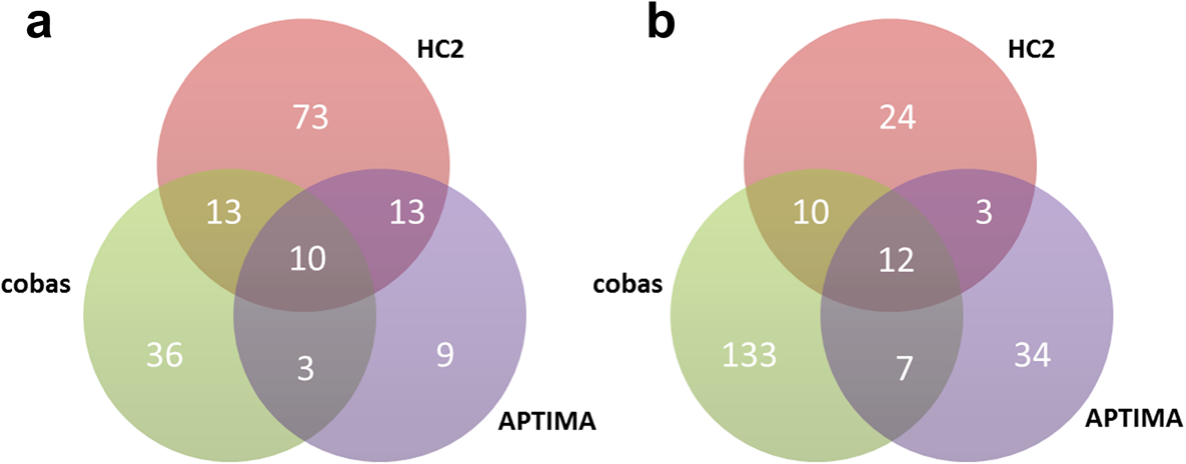
Inter-assay distribution of samples cross-reacting to low-risk genotypes **a** and unconfirmed genotypes **b**

Only 10 (6 %) of 157 samples cross-reacting to low-risk genotypes did so on all three assays (Fig. 1a). Of the 109 HC2 cross-reacting samples, 73 (67 %) were negative on cobas and APTIMA, and 24 of these involved genotype 66. Cobas had 36 (58 %) unique cross-reacting samples out of all 62, whereas APTIMA had 9 (26 %) out of 35. In total, 75 % of the 157 samples cross-reacting to low-risk genotypes were positive on only one assay. Among the 223 samples cross-reacting to unconfirmed genotypes, concordance was similarly low, with 12 (5 %) being positive on all three assays; 86 % of 223 samples were positive on only one (Fig. 1b).

One of the six samples cross-reacting to low-risk genotypes with histologically confirmed high-grade lesions was a cervical cancer, detected by all three assays (Table 3). Of the five CIN2/3, one was detected by HC2 and APTIMA (CIN 2), two by HC2 and cobas (one CIN2 and one CIN3), and two (both CIN3) only by HC2. One CIN3 was detected by all three assays but CLART detected no genotypes.

**Table 3:** Women with CIN2 or higher: Screening test results for cross-reacting samples

				HPV test result		
Case number	Grade of CIN	Cytology	Detected genotypes by CLART	HC2, relative light units per cut-off	cobas, cycle threshold	APTIMA, signal per cut-off
Cross-reactivity to low-risk genotypes						
1	Grade 2	Atypical	70, 71, 81, 84	Positive (cross-reacting), 9.55	Negative	Positive (cross-reacting), 3.44
2	Grade 2	Normal	53, 66, 83	Positive (cross-reacting), 3.58	Positive, other high-risk genotypes: 30.1	Negative
3	Grade 3	Low-grade	82	Positive (cross-reacting), 2.70	Positive (cross-reacting), genotype 16: 37.3	Negative
4	Grade 3	High-grade	42, 61	Positive (cross-reacting), 5.74	Negative	Negative
5	Grade 3	High-grade	82	Positive (cross-reacting), 11.06	Negative	Negative
6	Cervical cancer	High-grade	70	Positive (“cross-reacting”), 21.43	Positive (“cross-reacting”), genotype 18: 39.5	Positive (“cross-reacting”), 0.84
Cross-reactivity to unconfirmed genotypes						
7	Grade 3	Atypical	None	Positive (CLART negative), 2.14	Positive (CLART negative), other high-risk genotypes: 34.0	Positive (CLART negative), 14.92

### Multiple infections

As also described previously [7], the likelihood of an assay returning a positive test result increased with the number of genotypes present in the sample (Table 4). This was observed for all three assays.

**Table 4:** Breakdown of positive and negative test results on HC2, cobas, and APTIMA, by the number and risk level of HPV genotypes detected on CLART

Risk level of detected genotypes	Number of genotypes	HC2			cobas			APTIMA		
		Negative test result	Positive test result	Total	Negative test result	Positive test result	Total	Negative test result	Positive test result	Total
Only low-risk	1	410 (84 %)	79 (16 %)	489	390 (89 %)	47 (11 %)	437	412 (94 %)	25 (6 %)	437
	2	72 (80 %)	18 (20 %)	90	66 (85 %)	12 (15 %)	78	73 (94 %)	5 (6 %)	78
	3	18 (69 %)	8 (31 %)	26	16 (84 %)	3 (16 %)	19	16 (84 %)	3 (16 %)	19
	≥4	6 (60 %)	4 (40 %)	10	10 (100 %)	0 (0 %)	10	8 (80 %)	2 (20 %)	10
	Total	506 (82 %)	109 (18 %)	615	482 (89 %)	62 (11 %)	544	509 (94 %)	35 (6 %)	544
Only high-risk	1	245 (50 %)	242 (50 %)	487	143 (27 %)	396 (73 %)	539	319 (59 %)	220 (41 %)	539
	2	19 (10 %)	169 (90 %)	188	8 (5 %)	146 (95 %)	153	45 (29 %)	109 (71 %)	153
	3	3 (6 %)	45 (94 %)	48	1 (2 %)	53 (98 %)	54	13 (24 %)	41 (76 %)	54
	≥4	0 (0 %)	12 (100 %)	12	0 (0 %)	18 (100 %)	17	1 (6 %)	17 (94 %)	17
	Total	267 (36 %)	468 (64 %)	735	152 (20 %)	613 (80 %)	765	378 (49 %)	387 (51 %)	765
Low-risk and high-risk	2	72 (46 %)	86 (54 %)	158	42 (20 %)	162 (79 %)	205	106 (52 %)	98 (48 %)	205
	3	38 (25 %)	116 (75 %)	154	16 (10 %)	139 (90 %)	155	56 (36 %)	99 (64 %)	155
	≥4	19 (9 %)	196 (91 %)	215	2 (1 %)	207 (99 %)	210	46 (22 %)	163 (78 %)	210
	Total	129 (24 %)	398 (76 %)	527	60 (11 %)	508 (89 %)	568	208 (37 %)	360 (63 %)	568
No genotypes	0	3096 (98 %)	49 (2 %)	3145	2981 (95 %)	162 (5 %)	3143^a^	3089 (98 %)	56 (2 %)	3145
Total	–	–	–	5022	–	–	5020^a^	–	–	5022

^a^Two samples had an invalid test result on cobas. For both samples, HC2 and APTIMA test results were negative; CLART detected no genotypes; cytology on one sample was normal, and inadequate on the other sample

### Signal strength

**Fig. 2:**
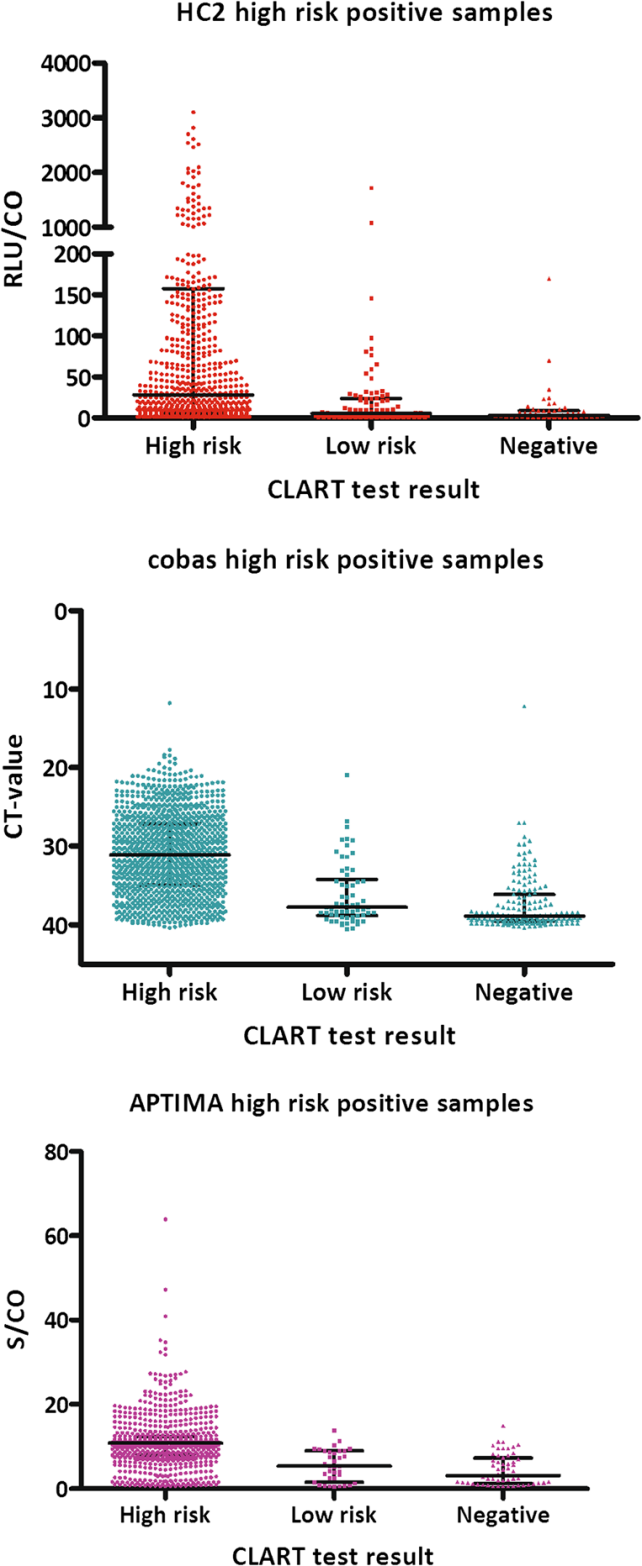
Signal strength of samples with a positive test result on HC2, cobas, or APTIMA. Test results are stratified by whether CLART detected at least one of the high-risk genotypes, only low-risk genotypes, or no genotypes. Medians with interquartile ranges (IQR). HC2: High-risk genotypes (*n* = 866), median = 28.1, IQR: 5.6 to 157.4. Low-risk genotypes (*n* = 109), median = 5.2, IQR: 2.2 to 24.1. No genotypes (*n* = 49), median = 3.0, IQR: 1.7 to 8.8. cobas: High-risk genotypes (*n* = 1121), median = 31.1, IQR: 27.1 to 35.0. Low-risk genotypes (*n* = 62), median = 37.8, IQR: 34.2 to 38.9. No genotypes (*n* = 162), median = 38.9, IQR: 36.2 to 39.5. APTIMA: High-risk genotypes (*n* = 747), median = 10.8, IQR: 8.0 to 12.4. Low-risk genotypes (*n* = 35), median = 5.4, IQR: 1.5 to 8.9. No genotypes (*n* = 56), median = 3.2, IQR: 1.3 to 7.3

For all three assays, the median signal strength was weaker for samples cross-reacting to low-risk genotypes than for samples with high-risk genotypes confirmed by CLART (Fig. 2). In samples cross-reacting to unconfirmed genotypes, the median signal strength levels tended to be lower than in samples cross-reacting to low-risk genotypes.

### Most frequently cross-reacting low-risk genotypes

For HC2, the most frequent cross-reacting infections were 66 (20), 70 (19), 53 (18), and 82 (14 %; Table 5). Several other genotypes also showed cross-reactivity, but in <10 % of all infections. On cobas, the frequent cross-reacting genotypes were 70 (19) and 61 (15 %). On APTIMA, although based on very low numbers, genotypes 70 (36), 62 (16), 61 (12), 82 (12), and 83 (12 %) dominated. Genotype distributions were similar in multiple infections, with genotypes 44, 71, and 72 detected only in cross-reacting samples with multiple infections. Genotypes 40, 43, 85, and 89, all very infrequent in this population [12], did not appear to be involved in cross-reactivity.

**Table 5:** Genotype distribution in samples cross-reacting to low-risk genotypes

Genotype	Phylogenetic clade	High-risk genotypes in the same phylogenetic clade	HC2		cobas		APTIMA	
			Single infections (%)	Multiple infections (%)	Single infections (%)	Multiple infections (%)	Single infections (%)	Multiple infections (%)
6	α10	None	2 (2.5 %)	5 (6.6 %)	3 (6.4 %)	4 (12.1 %)	0 (0.0 %)	2 (7.4 %)
11	α10	None	0 (0.0 %)	0 (0.0 %)	1 (2.1 %)	0 (0.0 %)	0 (0.0 %)	0 (0.0 %)
26	α5	51	0 (0.0 %)	0 (0.0 %)	1 (2.1 %)	0 (0.0 %)	0 (0.0 %)	0 (0.0 %)
42	α1	None	5 (6.3 %)	3 (3.9 %)	4 (8.5 %)	0 (0.0 %)	2 (8.0 %)	2 (7.4 %)
44	α10	None	0 (0.0 %)	3 (3.9 %)	0 (0.0 %)	2 (6.1 %)	0 (0.0 %)	0 (0.0 %)
53	α6	56	14 (17.7 %)	10 (13.2 %)	4 (8.5 %)	4 (12.1 %)	0 (0.0 %)	2 (7.4 %)
54	α13	None	2 (2.5 %)	2 (2.6 %)	2 (4.3 %)	2 (6.1 %)	0 (0.0 %)	1 (3.7 %)
61	α3	None	2 (2.5 %)	7 (9.2 %)	7 (14.9 %)	4 (12.1 %)	3 (12.0 %)	1 (3.7 %)
62	α3	None	2 (2.5 %)	5 (6.6 %)	4 (8.5 %)	2 (6.1 %)	4 (16.0 %)	3 (11.1 %)
66	α6	56	16 (20.3 %)	8 (10.5 %)	Not relevant	Not relevant	Not relevant	Not relevant
70	α7	18, 39, 45, 59, 68	15 (19.0 %)	8 (10.5 %)	9 (19.1 %)	3 (9.1 %)	9 (36.0 %)	4 (14.8 %)
71	α15	None	0 (0.0 %)	1 (1.3 %)	0 (0.0 %)	0 (0.0 %)	0 (0.0 %)	1 (3.7 %)
72	α3	None	0 (0.0 %)	1 (1.3 %)	0 (0.0 %)	1 (3.0 %)	0 (0.0 %)	1 (3.7 %)
73	α11	None	1 (1.3 %)	2 (2.6 %)	0 (0.0 %)	0 (0.0 %)	0 (0.0 %)	1 (3.7 %)
81	α3	None	3 (3.8 %)	4 (5.3 %)	3 (6.4 %)	2 (6.1 %)	1 (4.0 %)	2 (7.4 %)
82	α5	51	11 (13.9 %)	5 (6.6 %)	3 (6.4 %)	2 (6.1 %)	3 (12.0 %)	2 (7.4 %)
83	α3	None	2 (2.5 %)	7 (9.2 %)	3 (6.4 %)	3 (9.1 %)	3 (12.0 %)	2 (7.4 %)
84	α3	None	4 (5.1 %)	5 (6.6 %)	3 (6.4 %)	4 (12.1 %)	0 (0.0 %)	3 (11.1 %)
# Genotypes	–	–	79 (100 %)	76 (100 %)	47 (100 %)	33 (100 %)	25 (100 %)	27 (100 %)
# Samples	–	–	79	30	47	15	25	10

No woman had a cross-reacting sample on any of the three HPV assays because of genotypes 40 (α8), 43 (α8), 85 (α7), or 89 (α3)

On cobas, 5 % of samples with a positive test result on channel 16 alone contained only low-risk genotypes (Table 6). For channels 18 and other high-risk, the proportions were similar at 7 and 6 %, respectively. Of all samples cross-reacting to low-risk genotypes, 77 % (48/62) were on other high-risk channel alone. Similar proportions, in the range of 14-18 % for the three channels, were also found for cross-reactivity to unconfirmed genotypes.

**Table 6:** Genotype distribution in cross-reacting samples on cobas

Genotype	cobas test result							
	Genotype 16	Genotype 18	Other high-risk genotypes	16 and 18	16 and other high-risk	18 and other high-risk	16, 18, and other high-risk	Total
6	1	0	6	0	0	0	0	7
11	0	0	1	0	0	0	0	1
26	0	0	1	0	0	0	0	1
42	2	0	2	0	0	0	0	4
44	0	0	2	0	0	0	0	2
53	0	1	7	0	0	0	0	8
54	1	1	2	0	0	0	0	4
61	3	1	7	0	0	0	0	11
62	0	0	6	0	0	0	0	6
70	0	2	10	0	0	0	0	12
72	0	0	1	0	0	0	0	1
81	1	0	3	0	0	1	0	5
82	1	0	4	0	0	0	0	4
83	0	0	6	0	0	0	0	6
84	0	0	6	0	1	0	0	7
# Samples with a positive test result	168 (100 %)	56 (100 %)	858 (100 %)	6 (100 %)	167 (100 %)	71 (100 %)	19 (100 %)	1345 (100 %)
# Samples with only low-risk genotypes	8 (4.8 %)	4 (7.1 %)	48 (5.6 %)	0 (0.0 %)	1 (0.6 %)	1 (1.4 %)	0 (0.0 %)	62 (4.6 %)
# Samples with no detected genotype	26 (15.5 %)	10 (17.9 %)	116 (13.5 %)	0 (0.0 %)	8 (4.8 %)	2 (2.8 %)	0 (0.0 %)	162 (12.0 %)

No woman had a cross-reacting sample on cobas because of genotypes 40, 43, 71, 73, 85, or 89

### Effect of cross-reactivity on the proportions of women with false-positive test results

Cross-reactivity explained a measurable part of all false-positive test results (Table 7). In primary screening at 30–65 years, about one in four false-positive HPV test result was due to cross-reactivity. Had there been no cross-reactivity, 7.6 instead of 10.1 % of women would have had a false-positive test result on HC2. On cobas, this would have been 10.8 % instead of 14.5 %, and 6.2 % instead of 7.8 % on APTIMA.

**Table 7:** Effect of cross-reactivity on the proportion of women with positive and false-positive HPV test results

	HC2		cobas		APTIMA	
	*N* (%)	≥CIN2	*N* (%)	≥CIN2	*N* (%)	≥CIN2
Primary screening, 30–65 years (*n* = 2859)						
All positive test results	335 (11.7 %)	46	464 (16.2 %)	49	270 (9.4 %)	46
Samples with high-risk genotypes	262 (9.2 %)	45	357 (12.5 %)	48	221 (7.7 %)	45
Cross-reacting samples	73 (2.6 %)	1	107 (3.7 %)	1	49 (1.7 %)	1
False-positive test results, all (%)	289 (10.1 %)	–	415 (14.5 %)	–	224 (7.8 %)	–
False-positive test results, after exclusion of cross-reactivity (%)	217 (7.6 %)	–	309 (10.8 %)	–	176 (6.2 %)	–
Proportion of false-positive test results due to cross-reactivity	25 %	–	26 %	–	21 %	–
Referral population (*n* = 887)						
All positive test results	401 (45.2 %)	124	453 (51.1 %)	123	332 (37.4 %)	112
Samples with high-risk genotypes	345 (38.9 %)	117	410 (46.2 %)	120	316 (35.6 %)	109
Cross-reacting samples	56 (6.3 %)	7	43 (4.8 %)	3	16 (1.8 %)	3
False-positive test results, all (%)	277 (31.2 %)	–	330 (37.2 %)	–	220 (24.8 %)	–
False-positive test results, after exclusion of cross-reactivity (%)	228 (25.7 %)	–	290 (32.7 %)	–	207 (23.3 %)	–
Proportion of false-positive test results due to cross-reactivity	18 %	–	12 %	–	6 %	–

## Discussion

### General findings

In Danish routine SurePath samples, the patterns of HPV cross-reactivity for HC2 resembled those that were described previously. Cobas has been advertised as a HPV assay that does not cross-react to low-risk genotypes [20], whereas APTIMA’s package insert cites cross-reactivity to genotypes 26, 67, 70, and 82, which are phylogenetically related to high-risk genotypes [21]. In our study, however, both appeared to cross-react to low-risk genotypes from various phylogenetic clades including those that do not include high-risk genotypes.

The frequency of cross-reactivity to low-risk genotypes was most frequently observed on HC2. The number of samples in which none of the 35 CLART genotypes was detected was surprisingly high especially for cobas. When both types of cross-reactivity were combined, about a quarter of samples with a false-positive test result in primary screening at age 30–65 years appeared to be cross-reacting on any of the three assays.

For all three assays, the most frequently involved cross-reacting genotypes were 53, 61, 62, 70, 82, and for HC2 also genotype 66. Cross-reacting samples exhibited relatively weak signal strengths, and few were associated with ≥ CIN2. Cross-reactivity to low-risk genotypes was more frequent in young women, in abnormal cytology, and after previous abnormalities. Cross-reactivity to unconfirmed genotypes, on the other hand, tended to be more frequent in normal cytology. There were only a few samples that cross-reacted on all three assays, suggesting that cross-reactivity is driven by technology.

### Strengths and weaknesses

This is the first study that systematically evaluated cross-reactivity on three widely used assays in a split-sample study. It is, furthermore, the first independent study on cobas and APTIMA. We used consecutive, unselected, samples from women undergoing routine screening or follow-up of abnormalities. All testing was undertaken in the same laboratory by the same staff. This split-sample design helped eliminate variability in study populations and laboratory performance. Samples were stored in SurePath, a liquid-based cytology medium that is frequently used in Europe and the USA. Samples were heated to reverse the covalent bindings between genomic material and protein complexes induced by SurePath’s formaldehyde. This procedure renders the genetic material accessible for analysis [22]. We could determine the reason for sampling, enabling us to compare the frequency of cross-reactivity in the primary screening and referral populations. In line with our previous analyses [17], we again conclude that the data from referral populations cannot be generalised to the primary screening context.

Biological material can deteriorate or disintegrate upon storage. Prolonged storage could impact the data especially in samples with weak signal strength, a characteristic we observed in cross-reacting samples. Thus, using fresh samples, as we did in our study [12, 14, 16], may be the only reliable way to evaluate and compare the frequency of cross-reactivity. Moreover, in concordance with the protocol and by approval from the manufacturers, we diluted the original samples approximately 1:1. This can be seen as a weakness. However, all three assays rely on testing aliquots of 0.5–1 ml out of the typically 10 (SurePath) or 20 ml (ThinPrep) available from liquid-based cytology media. Hence, assay designs should be robust enough to handle sampling variability in terms of cellularity.

There is no internationally agreed standard genotyping assay, so the choice of a reference assay can be discussed [7, 11]. No HPV assay, with or without genotyping, seems to detect all targeted infections [17, 23]. CLART is a CE-IVD marked assay, not “research use only”, and is currently used in a number of regional European screening programmes. It has been evaluated in clinical settings [12, 24–28], and its analytical performance has been compared to, for example, linear array (LA) as part of the latest WHO HPV LabNet Proficiency Studies [29]. There, both assays showed a high analytical sensitivity for genotypes 16 and 18, even at low plasmid concentrations. CLART more often correctly detected genotypes 6, 11, 31, 33, 35, 51, 52, 58, 59, and 66 compared to LA, but the latter was better at detecting genotypes 45 and 56 at high plasmid concentrations. Finally, we chose CLART as a reference assay given that it reports the detected genotypes using a computer algorithm rather than manual reading. This enables a more reproducible and objective assay read-out.

In this study, CLART detected high-risk genotypes or genotype 66 in 27 % of all samples. This was comparable to the proportion in which cobas detected high-risk genotypes (27 %), and higher than the proportions detected by HC2 (20 %) and APTIMA (17 %). In a different study from our laboratory using data from 401 women with abnormal cytology [28], we also compared the detection of low-risk genotypes between CLART and LA. The detection of several genotypes found to be most frequently cross-reacting in the present study (53, 61, 66, and 70) was very similar, with an overall agreement of 98–99 %. For the other two most frequently cross-reacting genotypes (62 and 82), the level of agreement was slightly lower (96 and 93 %, respectively).

However, a general limitation of CLART is the relatively long amplicons generated from the modified PGMY09/11 primers, meaning that partially complete amplicons or unspecific amplifications are less likely to be reported as positive test findings compared to genotyping assays relying on shorter amplicons, such as LA. Furthermore, in our study CLART detected only a genotype 70 infection in one case of cervical cancer associated where cobas detected genotype 18 [15]. A CIN3 case was positive on all three evaluated assays but negative on CLART. The remaining five cases of CIN2/3 that were apparently missed by CLART were positive only on one or two of the evaluated assays. Given that the histology was read under routine circumstances, false-positive histology findings cannot be entirely ruled out [8].

Finally, the cross-reactivity estimates for APTIMA should be interpreted with respect to the fact that it detects HPV mRNA, whereas CLART detects HPV DNA. Therefore, APTIMA should ideally have been evaluated against an mRNA genotyping assay. However, no such assay exists.

### Comparison with the literature

Castle et al. [6] studied cross-reactivity patterns of HC2 probe B against the combined test results of MY09/11 Amplitaq DNA polymerase and Amplitaq Gold DNA polymerase in an unscreened population (*n* = 954). Of all single low-risk genotype infections, HC2 cross-reacted in 20 %, most frequently because of genotypes 11, 53, 61, 66, 67, 70, 71, and 81. Six (6 %) of 108 ≥ CIN3 were detected in cross-reacting samples, and 5 (5 %) in samples with no detected genotypes. In normal cytology, cross-reacting samples increased the sensitivity for high-grade CIN, whereas in abnormal cytology, they primarily decreased the specificity. Very similar results were found in the ALTS trial, using archived samples (collected in STM media or PreservCyt) from 3179 women with ASCUS/LSIL [7]. Cross-reactivity, assessed against the combined test results of line blot (a prototype for LA) and LA assays, was observed in 8 % of samples with a positive HC2 test result (4 % of all samples), whereas 2 % (1 %) had no detected genotypes on the reference assays. The most frequently involved genotypes were 66, 70, and 82, and the likelihood of cross-reactivity increased in multiple low-risk infections. Cross-reacting samples had weaker signals than samples with high-risk genotypes. Out of 272 ≥ CIN3, three (1 %) were from women with cross-reacting samples, and one (<1 %) from a woman with no detected genotypes. In a Guanacaste vaccination trial of women aged 18–25 years, Safaeian et al. [10] evaluated cross-reactivity of HC2 on ThinPrep samples against a highly analytically sensitive SPF-10 assay. While genotype 66 was not included in the analysis, 70 and 53 were most frequently involved in cross-reactivity. Low amounts of viral target input material and younger age were associated with cross-reactivity.

In the UK HPV screening trial ARTISTIC, Sargent et al. found very high proportions of cross-reacting samples at 20–64 years [11]. Of the 3773 HC2-positive ThinPrep samples, line blot assay detected only low-risk genotypes in 11 % (predominantly 53, 66, and 70), and no genotypes in additional 20 %. In approximately half of these samples, the signal strength was low, between 1 and 2 RLU/CO. In the Italian HPV screening trial NTCC, Gillio-Tos et al. [8] genotyped HC2-positive ThinPrep samples at 25–60 years using GP5+/GP6+ PCR with reverse line blot hybridisation, and, if no genotypes were found, also restriction fragment length polymorphism testing and sequencing. Relative cross-reactivity was 14 % (most frequently because of genotypes 66, 70, and 53), whereas in 7 % of HC2-positive samples no HPV DNA was detected. They suggested a role of the collection medium, and reported a higher probability of cross-reactivity for ThinPrep than for Specimen Transport Medium.

Overall, previous studies evaluated cross-reactivity for HC2 against a variety of genotyping assays, of which some were research versions and some have been discontinued. Several studies were undertaken on frozen samples stored in various sampling media. Nevertheless, our data are in line with previous observations. Independent evaluations of cross-reactivity for cobas and APTIMA have not been reported elsewhere, and as such represent valuable information for decision makers in choosing assays for screening purposes.

### Clinical and technical implications

Of the 175 CIN2/3 in our study, seven (4 %) were associated with cross-reactivity. The question is whether their detection and treatment prevented cervical cancer. It could be hypothesised that these cases were likely regressive. However, treatment of all high-grade CIN is recommended in Denmark, so this hypothesis cannot be evaluated using our data. For HC2, cross-reactivity to genotype 66 played an important role. Of all 52 single infections with this genotype, HC2 detected 16 (31 %). APTIMA and cobas were designed to detect this genotype. Yet, cobas detected only 31 (60 %), and APTIMA 11 (21 %) single-genotype 66 infections. Nevertheless, given that genotype 66 probably does not cause cervical cancer [5], the relatively inconsistent detection of this genotype unintentionally improves the clinical specificity of the two assays.

We propose three scenarios that may have contributed to cross-reactivity. Firstly, cross-reactivity to low-risk genotypes may have been generated by sequence homology in the assay amplification target region, whether that was *L1* (cobas), *E6/E7* (APTIMA), or whole HPV genome (HC2). In our data, all three assays showed rather extensive cross-reactivity to genotype 70. Genotype 70 shares phylogenetic clade (α7) with genotype 18. The latter is associated with adenocarcinomas and typically causes lesions characterised by low viral loads compared to e.g. genotype 16 [30–33]. It seems plausible that the assays may have been calibrated to detect genotype 18, but with an unintentional drawback of picking up same-clade low-risk genotypes. Within this context, the frequent cross-reactivity of HC2 to genotypes 53 and 66, and occasional cross-reactivity to genotypes 26 and 82, might be attributable to the fact that they share clades (α5, α6) with high-risk genotypes 51 and 56.

Secondly, cross-reactivity may have been caused by detection of non-specific, incomplete amplicons or by signal amplification probes hybridising to non-target sequences. Incomplete or unspecific amplifications would not hybridise efficiently to the array probes. When using technologies such as CLART with separate amplification and detection processes, this would most likely lead to detection of no genotype.

Thirdly, cross-reactivity may be generated by another aspect of the assays’ technical designs, in that specific or non-specific amplifications, otherwise below the positivity threshold, add up to push the total signal value above the manufacturer’s cut-off. This additive signal effect might explain the relatively high likelihood of cross-reactivity observed among younger women and women with abnormalities, as they tend to harbour the highest numbers of multiple infections. Consequently, assays with fewer genotype targets per channel or read-out could be speculated to be more precise from the analytical perspective, making the case for assays with genotyping beyond that of an individual detection of only genotypes 16 and 18.

## Conclusions

HC2, cobas and APTIMA all showed cross-reactivity which seemed to be driven primarily by the assays’ designs. A quarter of all false-positive test results in primary screening at ≥30 years cross-reacted. To obtain improved analytical and clinical performance, cross-reactivity should be addressed by optimising the assays. For now, cross-reactivity should be addressed in EU tenders, as this primarily technical shortcoming imposes additional costs on the screening programmes as well as risking the public’s view on the effectiveness of cervical screening.

## Abbreviations

ASCUS, Atypical squamous cells of undetermined significance; CIN, Cervical intraepithelial neoplasia; CT, Cycle threshold; HC2, hybrid capture II; HPV, Human papillomavirus; IQR, Interquartile range; LBC, Liquid based cytology; PCR, Polymerase chain reaction; RLU/CO, Relative light units/cut-off; S/CO, Signal/cut-off; STM, Specimen transport medium
